# Distinct Biogeographic Patterns for Archaea, Bacteria, and Fungi along the Vegetation Gradient at the Continental Scale in Eastern China

**DOI:** 10.1128/mSystems.00174-16

**Published:** 2017-02-07

**Authors:** Bin Ma, Zhongmin Dai, Haizhen Wang, Melissa Dsouza, Xingmei Liu, Yan He, Jianjun Wu, Jorge L. M. Rodrigues, Jack A. Gilbert, Philip C. Brookes, Jianming Xu

**Affiliations:** a Institute of Soil and Water Resources and Environmental Science, College of Environmental and Resource Sciences, Zhejiang University, Hangzhou, China; b Zhejiang Provincial Key Laboratory of Agricultural Resources and Environment, Hangzhou, China; c Department of Ecology and Evolution and Department of Surgery, University of Chicago, Chicago, Illinois, USA; d Department of Land, Air and Water Resources, University of California, Davis, Davis, California, USA; e Bioscience Division, Argonne National Laboratory, Lemont, Illinois, USA; f The Marine Biological Laboratory, Woods Hole, Massachusetts, USA; University of Tennessee

**Keywords:** Eastern China, edaphic factors, forest soil, historical processes, microbial diversity, vegetation zone

## Abstract

The natural forest ecosystem in Eastern China, from tropical forest to boreal forest, has declined due to cropland development during the last 300 years, yet little is known about the historical biogeographic patterns and driving processes for the major domains of microorganisms along this continental-scale natural vegetation gradient. We predicted the biogeographic patterns of soil archaeal, bacterial, and fungal communities across 110 natural forest sites along a transect across four vegetation zones in Eastern China. The distance decay relationships demonstrated the distinct biogeographic patterns of archaeal, bacterial, and fungal communities. While historical processes mainly influenced bacterial community variations, spatially autocorrelated environmental variables mainly influenced the fungal community. *Archaea* did not display a distance decay pattern along the vegetation gradient. Bacterial community diversity and structure were correlated with the ratio of acid oxalate-soluble Fe to free Fe oxides (Feo/Fed ratio). Fungal community diversity and structure were influenced by dissolved organic carbon (DOC) and free aluminum (Ald), respectively. The role of these environmental variables was confirmed by the correlations between dominant operational taxonomic units (OTUs) and edaphic variables. However, most of the dominant OTUs were not correlated with the major driving variables for the entire communities. These results demonstrate that soil archaea, bacteria, and fungi have different biogeographic patterns and driving processes along this continental-scale natural vegetation gradient, implying different community assembly mechanisms and ecological functions for archaea, bacteria, and fungi in soil ecosystems.

**IMPORTANCE** Understanding biogeographic patterns is a precursor to improving our knowledge of the function of microbiomes and to predicting ecosystem responses to environmental change. Using natural forest soil samples from 110 locations, this study is one of the largest attempts to comprehensively understand the different patterns of soil archaeal, bacterial, and fungal biogeography at the continental scale in eastern China. These patterns in natural forest sites could ascertain reliable soil microbial biogeographic patterns by eliminating anthropogenic influences. This information provides guidelines for monitoring the belowground ecosystem’s decline and restoration. Meanwhile, the deviations in the soil microbial communities from corresponding natural forest states indicate the extent of degradation of the soil ecosystem. Moreover, given the association between vegetation type and the microbial community, this information could be used to predict the long-term response of the underground ecosystem to the vegetation distribution caused by global climate change.

## Abstract (precis)

Understanding biogeographic patterns is a precursor to improving our knowledge of the function of microbiomes and to predicting ecosystem responses to environmental change. Using natural forest soil samples from 110 locations, this study is one of the largest attempts to comprehensively understand the different patterns of soil archaeal, bacterial, and fungal biogeography at the continental scale in eastern China. These patterns in natural forest sites could ascertain reliable soil microbial biogeographic patterns by eliminating anthropogenic influences. This information provides guidelines for monitoring the belowground ecosystem’s decline and restoration. Meanwhile, the deviations in the soil microbial communities from corresponding natural forest states indicate the extent of degradation of the soil ecosystem. Moreover, given the association between vegetation type and the microbial community, this information could be used to predict the long-term response of the underground ecosystem to the vegetation distribution caused by global climate change.

## INTRODUCTION

Eastern China experiences a continual natural vegetation gradient from tropical forest to boreal forest. There is growing awareness of the importance of soil microbiomes, including bacteria, archaea, and fungi, for regulating ecosystem services (1). Soil microbiomes perform the majority of soil carbon and nutrient biogeochemical transformations (2), control plant and animal population growth as decomposers, mutualists, or pathogens (3), and influence global climate change through greenhouse gas emissions (4). Soil microbiomes are extremely complex and diverse, with a large number of archaeal, bacterial, and fungal taxa being typically found in 1 g of soil (5). Numerous studies have established that microorganisms display spatial geographic patterns (6–8). Our recent study also confirmed clear biogeographic patterns for a cooccurrence relationship at the continental scale across Eastern China (9). However, while biogeographic patterns have been observed, little is known regarding the influence of the naturally occurring vegetation gradient on these patterns. Although these natural forest ecosystems have been extensively reduced by expanding cropland and urban area in the last 300 years (10), the ecosystem relicts preserved in nature reserves allow the reconstruction of historically widespread microbiomes (11). Given the relatively low anthropogenic influence on preserved natural forest ecosystems, we argue that it is possible to identify natural biogeographic patterns from microbiomes in forest reserves (12).

The biogeographic patterns of archaea, bacteria, and fungi have been investigated from local to global scales, but the exact mechanisms governing their distribution remain poorly understood (13). Microbial communities have been found to display different biogeographic patterns than plants and animals (14). Some studies showed that soil bacterial biogeographic patterns are controlled by soil pH at both regional and global scales (2, 12, 15). Other studies, however, suggest that bacterial spatial distribution patterns are associated with differences in carbon dynamics; e.g., the dominant *Verrucomicrobia* in prairie soils specialize in the degradation of recalcitrant carbon compounds (11). Globally, soil C/N ratios influence archaeal relative abundances, which are higher in soils with lower C/N ratios (16). Additionally, soil salinity, rather than temperature, is one of the principal driving forces responsible for the creation and maintenance of uncultured-*Archaea* distribution patterns at the global scale (17). A regional study along a steep precipitation gradient suggests that the compositions of archaeal and bacterial communities differ profoundly according to ecosystem type, which can be explained largely by the precipitation gradient combined with vegetation cover (18). Fungal richness is strongly and positively associated with soil pH and Ca concentration (2). However, processes operating at large spatial scales, such as dispersal limitation, were identified as first-order determinants of both regional species pools and the community composition of soil fungi at landscape scales across North American soil microbiomes (19). These apparently contradictory conclusions may arise through the differences in scale of the studies, since certain ecological processes might only be dominant at a particular scale (20). For example, dispersal barriers determine species pools at a large spatial scale, environmental conditions determine community composition in particular habitats at an intermediate spatial scale, and coexistence at a small spatial scale is determined by niche differences (19). Moreover, differences in methodological and theoretical frameworks make comparisons across studies difficult (14). Therefore, it is essential that consistent sampling and analytical methods should be applied to compare the biogeographic patterns of archaea, bacteria, and fungi under the same sampling regimes. Recent advances in DNA sequencing technology have permitted a more robust characterization of microbial biogeographic patterns (14). Despite this, to date, processes that determine the biogeographic patterns of soil archaea, bacteria, and fungi have not been investigated simultaneously.

In this study, we used large-scale, systematic sampling and soil analytical methods to investigate soil archaeal, bacterial, and fungal communities along a latitudinal gradient range from 18.9 to 48.7°N (over 3,000 km) across four continual vegetation types in Eastern China. These vegetation types included tropical seasonal forests (TSF), subtropical broad-leaved evergreen forests (SBEF), temperate deciduous broad-leaved forests (TDBF), and temperate mixed coniferous-broadleaf forests (TMCF) (see Table S1 in the supplemental material). Previous microbial biogeography studies in this region were largely carried out at local scales or on arable soils (21–23). To minimize the influence of land use on soil microbial communities (24), all soil samples in this study were collected from natural forest reserves, where the changes in soil microbial communities can represent unperturbed biogeographic patterns without obvious anthropogenic influence. The aim of this study was to determine and compare biogeographic patterns and drivers for archaeal, bacterial, and fungal communities at a continental scale. Our research questions were as follows. (i) Do biogeographic patterns differ for soil archaeal, bacterial, and fungal communities along this continual vegetation gradient? (ii) What are the determinant drivers for soil archaeal, bacterial, and fungal biogeographic patterns?

10.1128/mSystems.00174-16.6 TABLE S1  Vegetation types included in the study. Download TABLE S1, DOCX file, 0.04 MB. Copyright © 2017 Ma et al. 2017 Ma et al. This content is distributed under the terms of the Creative Commons Attribution 4.0 International license.

## RESULTS

### Changes in forest soil microbiota across the vegetation gradient.

**FIG 1:**
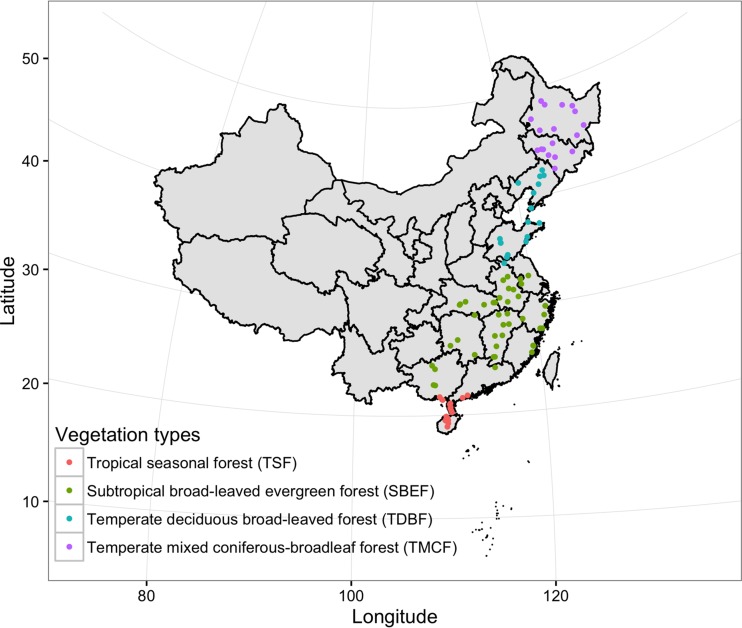
Locations and vegetation types of 110 sampling sites in China.

**FIG 2:**
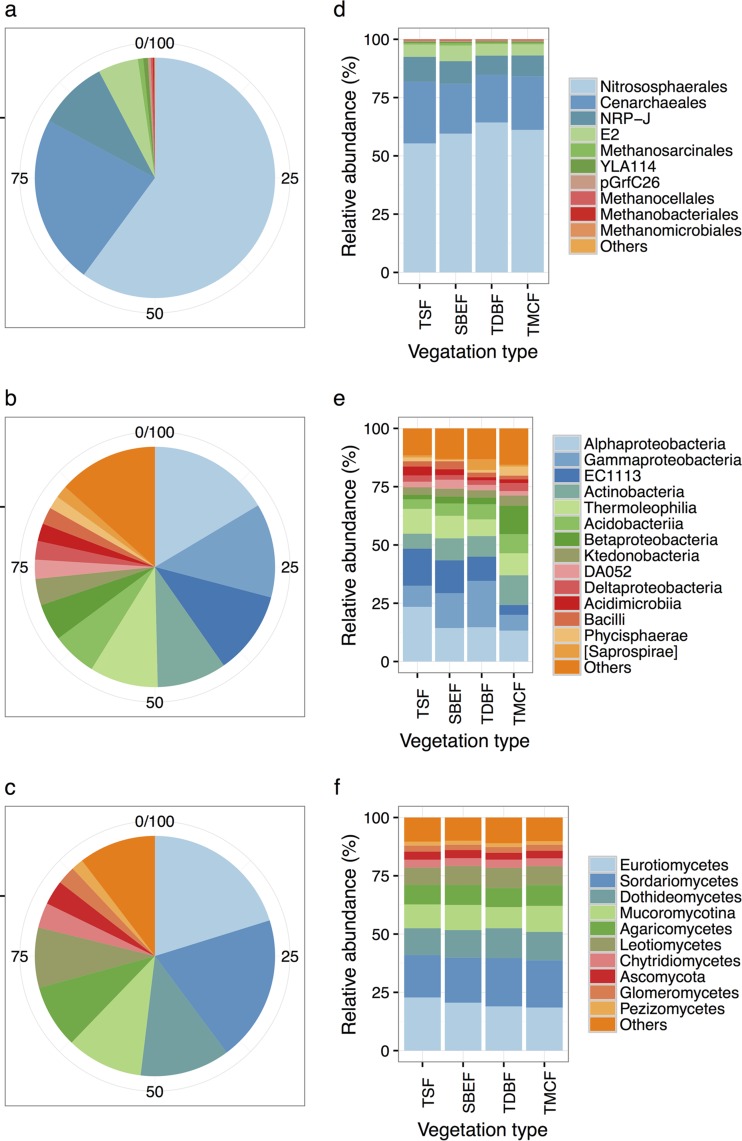
Composition profiles of archaeal, bacterial, and fungal communities in metacommunities (pie charts) and different vegetation types (bar charts).

We sequenced variable regions 3 to 5 (the V3–V5 region) of the archaeal 16S rRNA gene, the V1–V3 region of the bacterial 16S rRNA gene, and the V1–V3 region of the fungal 18S rRNA gene. After quality filtering, we obtained 144,706 archaeal sequences (1,316 ± 815 reads [mean ± standard deviation] per sample), 504,359 bacterial sequences (4,585 ± 1,354 reads per sample), and 470,872 fungal sequences (4,280 ± 2,133 reads per sample) from 110 distinct sampling sites (Fig. 1). At 97% sequence identity, a total of 3,366 operational taxonomic units (OTUs) were detected for the archaeal community, 57,561 for the bacterial community, and 12,862 for the fungal community. Nine samples with less than 300 archaeal sequences were removed from the archaeal community analysis. A majority of the archaeal sequences belonged to the orders *Nitrososphaerales* (59.7%), *Cenarchaeales* (22.5%), and NRP-J (5.6%) of the phylum *Thaumarchaeota* (92.3%) and subgroup E2 (5.8%) of the phylum *Euryarchaeota* (7.1%) (Fig. 2a). Members of the bacterial taxa *Alphaproteobacteria* (16.4%), *Gammaproteobacteria* (12.7), EC1113 (11.2%), *Actinobacteria* (9.4%), *Thermoleophilia* (9.2%), *Acidobacteria* (6.0%), and *Betaproteobacteria* (5.0%) comprised the largest proportion of sequences (Fig. 2b). The most abundant fungal classes belonged to the classes Eurotiomycetes (20.2%), Sordariomycetes (19.6%), Dothideomycetes (12.1%), and Leotiomycetes (8.1%) of the phylum Ascomycota (73.7%), class Mucoromycotina (10.3%) of the phylum Mucoromycota (10.3%), and class Agaricomycetes (8.5%) of the phylum Basidiomycota (8.5%) (Fig. 2c). Overall, our OTU-level classification revealed that 128 (3.8%) archaeal OTUs and 6,021 (10.5%) bacterial OTUs exhibited >97% identity to 16S rRNA gene sequences in the Greengenes database (13_8 release). Likewise, 2,762 (21.5%) fungal OTUs exhibited >97% identity to 18S rRNA gene sequences in the SILVA database (version 111).

The dominant archaeal, bacterial, and fungal phylogenetic groups were present in soils supporting all vegetation types, but their relative proportions varied across the vegetation gradient. For archaeal taxa (Fig. 2d), the relative abundance of *Nitrososphaerales* was highest in TDBF (64.3%) and lowest in TSF (55.3%). Conversely, the relative abundances of *Cenarchaeales* and NRP-J were highest in TSF (26.4% and 10.7%) and lowest in TDBF (20.4% and 8.3%). Members of E2 were most abundant in SBEF (6.7%). For bacterial taxa (Fig. 2e), the relative abundances of *Alphaproteobacteria* were highest in TSF (31.3%) and lowest in TMCF (23.8%). The relative abundances of *Thermoleophilia* ranged from 10% in TSF to 14.4% in TMCF. *Acidobacteria*, *Ktedonobacteria*, and ABS-6 were abundant in TDBF (8.3, 2.7, and 1.4% relative abundances, respectively), SBEF (7.9, 3.5, and 4%, relative abundances, respectively), and TSF (7.8, 2, and 1.7% relative abundances, respectively). For fungal taxa (Fig. 2f), members of Eurotiomycetes were the most abundant in TSF (36.8%). The relative abundances of Sordariomycetes (27.5%), Dothideomycetes (13.6%), Leotiomycetes (11.3%), Cryptomycotina (3.8%), and Chytridiomycetes (1.8%) were highest in TMCF. Likewise, the relative abundances of Mucoromycotina, Agaricomycetes, and Glomeromycetes were highest in TMCF (17.5, 7.4, and 1.6%, respectively).

### Geographic patterns of microbial community diversity.

**FIG 3:**
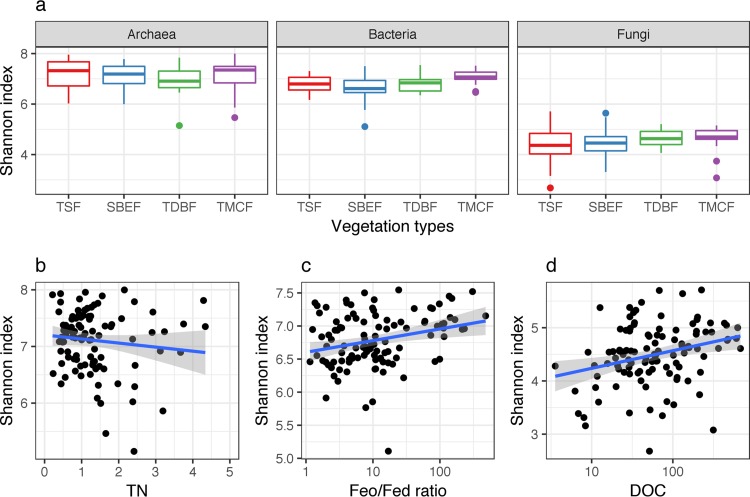
Shannon diversity index values for archaeal, bacterial, and fungal communities. (a) Distributions of archaea, bacteria, and fungi in tropical seasonal forests (TSF), subtropical broad-leaved evergreen forests (SBEF), temperate deciduous broad-leaved forests (TDBF), and temperate mixed coniferous-broadleaf forests (TMCF) and relationships between (b) Shannon index values of archaeal community and total nitrogen (TN), (c) bacterial community and amorphous iron/free iron ratio (Feo/Fed ratio), and (d) fungal community and dissolved organic carbon (DOC). The boxes show the distribution of values. The lower and upper hinges correspond to the first and third quartiles. The upper and lower whiskers extend from the hinge to the largest value no further than 1.5 times of the interquartile range from the upper and lower hinges, respectively. The outlier points are the data beyond the end of the whiskers.

We used the Shannon index to measure microbial alpha-diversity in each of the soil samples (101 samples for archaea and 110 for bacteria and fungi). The differences between the Shannon index values for the different vegetation types were not significant for archaea (*F* = 0.98, *df* = 3, *P* = 0.41) and fungi (*F* = 1.29, *df* = 3, *P* = 0.28). However, significant differences in the bacterial Shannon index values were observed for the different vegetation types (*F* = 5.32, *df* = 3, *P* = 0.002) (Fig. 3a). The mean bacterial Shannon index values in the SBEF were significantly lower than those observed in the TMCF (*P* < 0.001, Tukey honestly significant difference [HSD], 95% confidence).

We performed multiple regression modeling (MRM) and structural equation modeling (SEM) to test for the effects of environmental variables, including edaphic and climatic factors, on the Shannon index values. The archaeal Shannon index values were not significantly affected by any of the environmental variables. However, total nitrogen (TN) explained 16% of the archaeal Shannon index variation (*F* = 3.36, *df* = 99, *P* = 0.07 for MRM; *P* = 0.02 in SEM) (Fig. 3b; see also Fig. S1a in the supplemental material). The ratio of acid oxalate-soluble Fe to free Fe oxides (Feo/Fed ratio) was the strongest predictor of bacterial Shannon index values (*F* = 12.85, *df* = 108, *P* < 0.001 for MRM) (Fig. 3c) and explained 12% of the bacterial Shannon index variation (*P* = 0.05 in SEM) (see Fig. S1b). The fungal Shannon index values were significantly influenced by dissolved organic carbon (DOC) (*F* = 5.88, *df* = 108, *P* = 0.017) (Fig. 3d). This observation was also reflected in our SEM results (*P* = 0.05), where 21% of the variation in the fungal Shannon index values was explained by DOC (see Fig. S1c).

10.1128/mSystems.00174-16.1 FIG S1  Relationships among variables in SEM models for *archaea* (a), *bacteria* (b), and fungi (c). Div, Shannon diversity index; pH, soil pH; TN, total nitrogen concentrations; MAAT, mean annual air temperature; MAP, mean annual precipitation; Fed, free iron; FFR, amorphous iron/free iron ratio (Feo/Fed); AK, available potassium; TDN, total dissolved nitrogen; Ald, free aluminum; DOC, dissolved organic carbon; Clay, proportion of clay. Download FIG S1, EPS file, 0.2 MB. Copyright © 2017 Ma et al. 2017 Ma et al. This content is distributed under the terms of the Creative Commons Attribution 4.0 International license.

**FIG 4:**
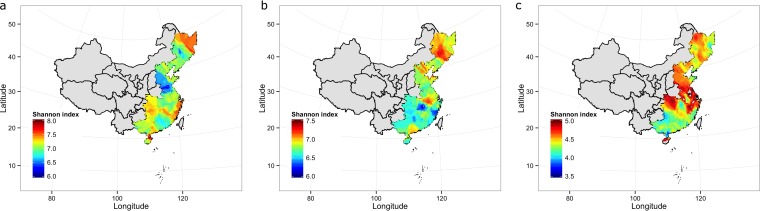
Spatial mapping of Shannon index values for archaeal (a), bacterial (b), and fungal communities (c) across sampling regions using kriging interpolation.

To extend our results beyond the 110 soil samples directly assayed, we constructed spatial maps of the alpha-diversities of soil microbial communities for whole sampling regions using a kriging interpolation approach (Fig. 4). The predicted maps showed that soils from the central region of China had lower archaeal diversities but higher bacterial and fungal diversities. Soils from the northern region had higher bacterial and fungal diversities than those in the southern regions. In addition, bacterial and fungal diversities displayed different spatial patterns in the northern regions. All of the predicted diversity distributions for archaea, bacteria, and fungi exhibited nonrandom spatial patterns. However, geographic distance had no significant effect on Shannon index values (the Mantel *r* values were 0.02 for archaea [*P* = 0.26], 0.01 for bacteria [*P* = 0.47], and 0.02 for fungi [*P* = 0.26]).

**FIG 5:**
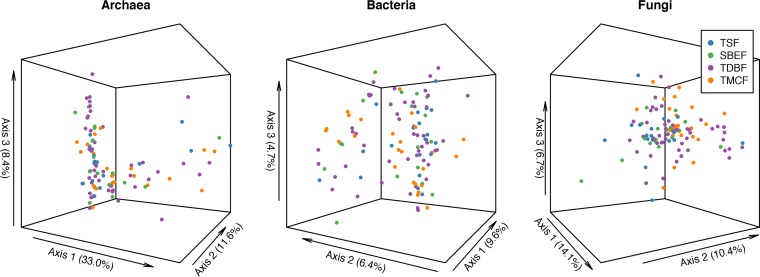
Ordination of microbial communities. The Bray-Curtis dissimilarity distances are represented using PCoA ordination for archaeal, bacterial, and fungal communities in tropical seasonal forests (TSF), subtropical broad-leaved evergreen forests (SBEF), temperate deciduous broad-leaved forests (TDBF), and temperate mixed coniferous-broadleaf forests (TMCF).

To evaluate changes in microbial composition across the four vegetation types, we used principal coordinate analysis (PCoA) to represent pairwise Bray-Curtis dissimilarities across soil microbial community profiles (Fig. 5). We also used permutational multivariate analysis of variance (PERMANOVA) to assess significant differences in microbial community compositions across the vegetation types. While the archaeal communities were not significantly different (*R*^*2*^ = 0.03, *P* = 0.68) across the different vegetation types, the bacterial (*R*^*2*^ = 0.055, *P* = 0.001) and fungal communities (*R*^*2*^ = 0.054, *P* < 0.001) showed significant differences according to vegetation type. We then utilized linear fitting to reveal the effects of environmental variables on compositional variations. The archaeal community composition was significantly affected by mean annual air temperature (MAAT) (*R*^*2*^ = 0.074, *P* = 0.024) (see Table S2 in the supplemental material). The bacterial community composition was dominantly affected by the Feo/Fed ratio (*R*^*2*^ = 0.13, *P* = 0.002), Fed (*R*^*2*^ = 0.08, *P* = 0.01), total dissolved nitrogen (*R*^*2*^ = 0.07, *P* = 0.02), humic acid (*R*^*2*^ = 0.06, *P* = 0.04), and available potassium (*R*^*2*^ = 0.06, *P* = 0.04) (see Table S3). The fungal community composition was dominantly affected by free aluminum (Ald) (*R*^*2*^ = 0.142, *P* = 0.001), mean annual precipitation (MAP) (*R*^*2*^ = 0.15, *P* = 0.002), MAAT (*R*^*2*^ = 0.14, *P* = 0.002), and soil pH (*R*^*2*^ = 0.11, *P* = 0.01) (see Table S4). These observations were confirmed by constrained correspondence analysis (CCA), where the Feo/Fed ratio and Ald were the most important variables associated with changes in bacterial and fungal communities, respectively (see Fig. S2 to S4).

10.1128/mSystems.00174-16.2 FIG S2  Constrained correspondence analysis of the archaeal community. Ald, free aluminum; HA, free aluminum; MAAT, mean annual air temperature. Download FIG S2, EPS file, 0.3 MB. Copyright © 2017 Ma et al. 2017 Ma et al. This content is distributed under the terms of the Creative Commons Attribution 4.0 International license.

10.1128/mSystems.00174-16.3 FIG S3  Constrained correspondence analysis of the bacterial community. Fed, free iron; Feo/Fed ratio, amorphous iron/free iron ratio; HA, humic acid; MAAT, mean annual air temperature; MAP, mean annual precipitation; pH, soil pH; TDN, total dissolved nitrogen. Download FIG S3, EPS file, 1.7 MB. Copyright © 2017 Ma et al. 2017 Ma et al. This content is distributed under the terms of the Creative Commons Attribution 4.0 International license.

10.1128/mSystems.00174-16.4 FIG S4  Constrained correspondence analysis of the fungal community. Ald, free aluminum; Alo, amorphous aluminum; Clay, proportion of clay; Fed, free iron; HA/FA ratio, humic acid/fulvic acid ratio; MAAT, mean annual air temperature; MAP, mean annual precipitation; pH, soil pH. Download FIG S4, EPS file, 1.8 MB. Copyright © 2017 Ma et al. 2017 Ma et al. This content is distributed under the terms of the Creative Commons Attribution 4.0 International license.

10.1128/mSystems.00174-16.7 TABLE S2  Summary of relationships between environmental factors and archaeal communities. Correlations with numerical environmental variables (*r*^*2*^) were obtained by fitting linear trends to the PCoA ordination, and significance (*P*) was determined by permutation (perm = 9,999). Download TABLE S2, DOCX file, 0.1 MB. Copyright © 2017 Ma et al. 2017 Ma et al. This content is distributed under the terms of the Creative Commons Attribution 4.0 International license.

10.1128/mSystems.00174-16.8 TABLE S3  Summary of relationships between environmental factors and bacterial communities. Correlations with numeric environmental variables (*r*^*2*^) were obtained by fitting linear trends to the PCoA ordination, and significance (*P*) was determined by permutation (perm = 9,999). Download TABLE S3, DOCX file, 0.1 MB. Copyright © 2017 Ma et al. 2017 Ma et al. This content is distributed under the terms of the Creative Commons Attribution 4.0 International license.

10.1128/mSystems.00174-16.9 TABLE S4  Summary of relationships between environmental factors and fungal communities. Correlations with numeric environmental variables (*r*^*2*^) were obtained by fitting linear trends to the PCoA ordination, and significance (*P*) was determined by permutation (perm = 9,999). Download TABLE S4, DOCX file, 0.1 MB. Copyright © 2017 Ma et al. 2017 Ma et al. This content is distributed under the terms of the Creative Commons Attribution 4.0 International license.

**FIG 6:**
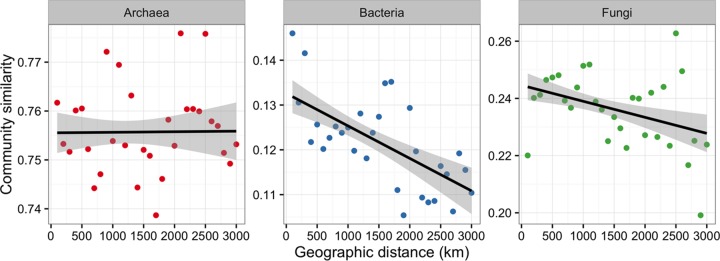
Relationships between the Bray-Curtis similarities of archaeal, bacterial, and fungal communities and geographic distance. The lines represent the linear regression results. The shaded areas show the 95% confidence interval.

To identify additional spatial sources of variation in the microbial communities, we examined the correlations between community similarity and spatial distance matrices. When examining entire data sets, we found that geographic distance correlated positively with bacterial (*R*^*2*^ = 0.06, *P* < 0.001) and fungal (*R*^*2*^ = 0.09, *P* < 0.001) community dissimilarities. In contrast, archaeal community dissimilarities were not correlated with geographic distance (*R*^*2*^ = −0.02, *P* = 0.105) (Fig. 6). The Mantel test results showed that dissimilarities in bacterial communities were significantly correlated with geographic distance (*P* = 0.012) and dissimilarities in fungal communities were significantly correlated with environmental variables (*P* = 0.010) and marginally significantly correlated with geographic distance (*P* = 0.051). The partial Mantel test results indicated that bacterial and fungal communities were primarily governed by spatial distance when the variation associated with environmental variables was removed (*P* = 0.011 for bacteria, and *P* = 0.016 for fungi) (Table 1).

**TABLE 1:** Mantel and partial Mantel test results for the correlation between community similarity and environmental and geographic distance

Effect of	Controlling for	Mantel statistic *r* (*P* value) for^a^:		
		*Archaea*	*Bacteria*	Fungi
Geographic distance		−0.015 (0.657)	0.059 (0.012*)	0.055 (0.051)
Environmental variables		−0.044 (0.738)	0.010 (0.398)	0.109 (0.010**)
Geographic distance	Environmental variables	0.012 (0.370)	0.054 (0.011*)	0.056 (0.016*)
Environmental variables	Geographic distance	−0.043 (0.738)	0.026 (0.234)	0.012 (0.314)

a Statistical significance was tested based on 9,999 permutations. *, *P* < 0.05; **, *P* < 0.01.

### Associations between dominant OTUs and environmental factors.

**FIG 7:**
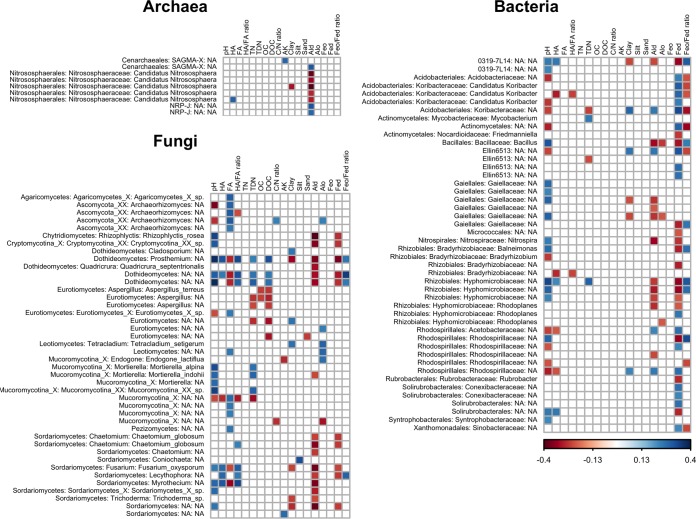
Correlations between dominant microbial OTUs and edaphic variables. Colors indicate the Spearman’s correlation coefficients, as shown in the key. Dominant OTUs uncorrelated with any edaphic variables are not displayed. The order, family, and genus names for the corresponding OTUs are shown to the left of the grids; “NA” indicates unclear classification at the corresponding taxonomic level. pH, soil pH; HA, humic acid; FA, fulvic acid; HA/FA ratio, humic acid/fulvic acid ratio; TN, total nitrogen; TDN, total dissolved nitrogen; OC, organic carbon; DOC dissolved organic matter; C/N ratio, carbon/nitrogen ratio; AK, available potassium; Clay, proportion of clay; Silt, proportion of silt; Sand, proportion of sand; Ald, free aluminum; Alo, amorphous aluminum; Feo, amorphous iron; Fed, free iron; Feo/Fed ratio, amorphous iron/free iron ratio.

The responses of the different taxa to different environmental variables may vary. Therefore, we calculated the Spearman correlation coefficients between environmental variables and dominant OTUs in archaeal, bacterial, and fungal communities (Fig. 7; see also Fig. S5 in the supplemental material). Dominant OTUs were defined as those with relative abundances above 0.1%. These included 42 archaeal OTUs, 85 bacterial OTUs, and 108 fungal OTUs. Most of the dominant microbial OTUs were not associated with any of the environmental variables measured (see Fig. S5). Fifty-four dominant bacterial OTUs (63.5% of the dominant bacterial OTUs) mainly correlated with the Feo/Fed ratio, Fed, amorphous aluminum (Alo), and pH. Clay content, DOC, and Ald were the most crucial environmental variables for 43 fungal OTUs (39.8% of the dominant fungal OTUs). Soil Ald was the major edaphic variable closely correlated with 9 dominant archaeal OTUs (21.4% of the dominant fungal OTUs). The dominant microbial OTUs from the same taxonomic group had similar responses to environmental variables. Ald was negatively correlated with archaeal OTUs belonging to the genera *Nitrososphaera* but positively correlated with the archaeal OTUs belonging to the NRP-J group. One archaeal OTU belonging to the SAGMA-X group correlated positively with available potassium. The soil Fed concentrations were positively correlated with the bacterial OTUs belonging to the orders *Acidobacteriales*, *Actinomycetales*, and *Solirubrobacterales* but negatively correlated with the OTUs belonging to the order *Rhizobiales*. The bacterial OTUs belonging to the order *Gaiellales* were not correlated with soil Fed concentrations but were either positively correlated with soil pH or negatively correlated with soil Ald concentrations. The soil Ald concentrations were negatively correlated with the fungal OTUs belonging to the Dothideomycetes and Sordariomycetes. The fungal OTUs belonging to the *Archaeorhizomyces* and Mucoromycotina, however, were correlated with either soil fulvic acid (FA) concentrations or soil pH rather than soil Ald concentrations. The fungal OTUs belonging to the Eurotiomycetes were negatively correlated with soil DOC concentrations rather than soil Ald concentrations.

10.1128/mSystems.00174-16.5 FIG S5  Correlations between dominant microbial OTUs and edaphic variables. Colors indicate the Spearman’s correlation coefficients, as shown in the key. The order, family, and genus names for the corresponding OTUs are shown beside the grids; “NA” indicates unclear classification at the corresponding taxonomic level. pH, soil pH; HA, humic acid; FA, fulvic acid; HA/FA ratio, humic acid/fulvic acid ratio; TN, total nitrogen; TDN, total dissolved nitrogen; OC, organic carbon; DOC, dissolved organic matter; C/N ratio, carbon/nitrogen ratio; AK, available potassium; Clay, proportion of clay; Silt, proportion of silt; Sand, proportion of sand; Ald, free aluminum; Alo, amorphous aluminum; Feo, amorphous iron; Fed, free iron; Feo/Fed ratio, amorphous iron/free iron ratio. Download FIG S5, TIF file, 1.9 MB. Copyright © 2017 Ma et al. 2017 Ma et al. This content is distributed under the terms of the Creative Commons Attribution 4.0 International license.

## DISCUSSION

Understanding biogeographic patterns requires characterization of the contributions of contemporary environmental factors versus those made by historical events. Given that distance decay of community similarity indicates the influence of historical factors (5), the different influences of geographic distance on archaeal, bacterial, and fungal community dissimilarities (Fig. 6) imply a discrepancy in the historical effects upon the different taxa. The distance decay pattern of bacterial community dissimilarity is consistent with previous observations based on high-throughput sequencing (25) but not with those using relatively low-resolution community profiling tools, such as terminal restriction fragment length polymorphism (T-RFLP) (9). Likewise, the correlation between geographic distance and fungal community dissimilarity agrees with previous observations of the global distribution of soil fungi (2) and protists (26). We did not observe a distance decay pattern for archaea, as previously reported for trophic lake sediments (27), or for ammonium-oxidizing archaea, as previously reported for the agricultural soils of Eastern China (28). However, spatial distance was also not a significant predictor of archaeal community composition in subpolar and arctic waters (29). The distance decay pattern of microbial community dissimilarity is generated by selection effects and drift, counteracted by dispersal, and modified by mutation (14). The effects of mutation cannot be considered in this data set, due to the highly conserved nature of the 16S and 18S rRNA marker genes (30). We found that the archaeal community did not show distance decay at the continental scale and was not significantly influenced by the selection effects of local environmental variables. Archaeal species represent a low proportion of total microbial communities and can be grouped into the rare biosphere (31). When relative abundance is low, selection is ineffective, and neutral forces shape rare biosphere assemblies (32). These assemblies may persist in the environment for a long time and exhibit different biogeographic patterns than groups that are more abundant. However, the lack of a distance decay pattern for archaea might only be due to their cosmopolitan ability to thrive in a variety of habits or domain-level resolutions which obscured meaningful correlations at lower taxonomic levels. The spatial structures of bacteria and fungi may have resulted from autocorrelated environmental variables or from dispersal limitation. In our study, bacterial community similarities were not correlated with environmental variables regardless of whether the test was controlled by the geographic distance or not, indicating that the distance decay pattern for bacterial communities related to historical dispersal limitation. The Mantel test results suggested a selection effect of environmental variables on fungal communities (Table 1). However, this correlation of environmental variables disappeared when the geographic distance was controlled in the partial Mantel test. Accordingly, the distance decay patterns of fungi can be predominantly attributed to the autocorrelated environmental variables. The contribution of historical processes represented by the distance effect may be overestimated, as a spurious distance effect will be found if some spatial autocorrelated environmental variables are not measured, such as climatic conditions, countless edaphic variables, and the disregarded interactions within microbial communities. The correlation between different compositions in a community influences microbial distributions and also displays biogeographic patterns (33).

Evidence for distinct biogeographic patterns of the three microbial taxa was also witnessed through the differentiation of community diversity across vegetation types (Fig. 3a). Consistent changes in the composition of the dominant taxa may result in differences in the diversity of microbiomes across environmental gradients. Our results suggested that the diversity of archaeal, bacterial, and fungal communities was controlled by different environmental variables (Fig. 3b to d). The strong positive influence of the Feo/Fed ratio on bacterial community diversity and structure is related to its influence on bacterial succession during soil development (34). The Feo/Fed ratio has been used for characterizing Fe oxide crystallinity and measuring the proportion of amorphous Fe in total Fe oxides (35). In global and regional soils, pH was identified as a major environmental variable that explained bacterial community diversity and composition (1, 15, 36). Fierer and Jackson (1) reported that pH is probably masking another environmental variable driving community assembly. The close association between the Feo/Fed ratio and soil pH (35) suggests that the effects of soil pH on bacterial community diversity and composition may be related to the soil Feo/Fed ratio. The direct influence of DOC on fungal diversity agrees with the observed positive correlation between ectomycorrhizal fungal (EMF) diversity and DOC (37). The global fungal diversity is strongly affected by soil pH and Ca concentration (2). Notably, the effect of Ca on fungal community diversity was attributed to the positive influence of exchangeable Ca on the turnover rate of soil organic matter (38). The large influence of soil Ald on fungal community diversity suggests that the community diversity may be controlled by available aluminum, which increases as soil pH declines (39). The previously reported influence of soil pH on fungal community structure may be attributed to aluminum toxicity. The tolerance of fungi for aluminum toxicity varies with different phylogenetic groups (40). The marginally significant influence of TN on archaeal diversity may be associated with the dominance of *Nitrososphaerales*, which can drive nitrification in soils (41).

The nonrandom spatial variation of community composition (Fig. 2) and diversity (Fig. 3) provides further evidence that archaea, bacteria, and fungi display different biogeographic patterns. This was clearly observed through the influence of vegetation type on the relative abundance of dominant taxa. The variation in the relative abundances of *Nitrososphaerales* indicated that ammonium oxidation activity is higher in temperate regions (TDBF and TMCF) than in tropical regions (TSF and SBEF). Members of *Nitrososphaerales* play a crucial role in ammonia-oxidizing processes in soil (42). The tendencies of *Cenarchaeales* and NRP-J suggest that those archaeal groups may respond negatively to ammonium oxidation activities. The members of *Cenarchaeales* have yet to be cultured (43), but denitrification genes were found in the genomes of *Cenarchaeales* based on metagenomic approaches (44). The uncultured NRP-J in *Crenarchaeota* were identified from soils, but their functions were unknown (45). The dominant bacterial taxa identified as part of this study were previously reported as dominant taxa in soils globally (46). In addition to this, we found different distribution patterns for several of these dominant bacterial taxa between tropical (TSF and SBEF) and temperate regions (TDBF and TMCF). For example, the relative abundances of the classes *Acidobacteria* and *Betaproteobacteria* were higher in the temperate regions than in tropical regions, but vice versa the relative abundances of the classes EC 1113 and *Thermoleophilia* were higher in tropical regions than in temperate regions. Although these groups are ubiquitous in soil microbiomes and our understanding of their distribution is continually increasing, their functions remain largely unexplored. Interestingly, it has been shown that the profiles of dominant fungal groups in this study are divergent with previously reported fungal communities in soils globally (2), where half of the sequences were classified as Agaricomycetes. However, Agaricomycetes sequences constituted less than 10% of all fungal sequences in our study. This discrepancy may be attributed to the different resolutions offered by the internal transcribed spacer (ITS) and 18S rRNA genes. The observed biogeographic patterns of dominant fungal classes suggested a differentiation of dominant fungal groups across the different vegetation types. Since most fungal species are uncultured as yet, their characteristics and functions are poorly understood. Consequently, multiple alternative interpretations can be made from their biogeographic patterns.

The most important environmental variables for the dominant microbial OTUs, including Feo/Fed ratio, Ald, and DOC (Fig. 7), were also identified as the best predictors of community structure in CCA analysis (see Fig. S2 to S4 in the supplemental material). Recently, studies of bacterial and eukaryotic communities revealed distinct patterns for dominant and rare biospheres and identified similar controlling factors between dominant OTUs and the entire community (47, 48). Our analysis, however, highlights that the influence of less important environmental variables cannot be neglected. Although not important drivers for whole communities, those environmental variables were the dominant drivers for specific taxonomic groups, e.g., Ald for *Gaiellales*, FA for *Archaeorhizomyces* and Mucoromycotina, and DOC for Eurotiomycetes. The global fungal biogeographic pattern indicated that, in specific regions, abiotic conditions (such as soil pH and climatic conditions) have probably stimulated evolutionary radiations in certain geographic areas (2). Therefore, the dominant OTUs associated with particular environmental variables that have smaller contributions to the entire community may be stimulated by these environmental variables in specific regional areas. Furthermore, dominant OTUs whose distribution patterns cannot be explained by any measured environmental variable may lack obvious biogeographic patterns or may be controlled by environmental variables that were not measured in this study. Another explanation for this result may be that some OTUs have shorter phylogenetic histories and have not had sufficient time for long-distance dispersal (49). Although the correlated environmental variables varied in the same taxa, the dominant microbial OTUs belonging to the same taxonomic groups had similar responses to environmental variables, suggesting that the responses of microbes to environmental variables associate with more highly resolved phylogenetic relationships.

We showed distinct biogeographic patterns for soil archaeal, bacterial, and fungal communities in the natural forest sites across the vegetation gradient in Eastern China. The continental-scale distributions of soil archaea, bacteria, and fungi were correlated with different edaphic variables. While the similarity of the archaeal communities was homogenous along geographic distance, the distance decay of bacterial and fungal community similarities was explained by dispersal limitation and the spatially autocorrelated environmental variables, respectively. These results imply distinct mechanisms for shaping the biogeographic patterns of different microbial taxa. The microbial biogeographic patterns inferred from the natural forest soils could provide more reliable evidence for understanding underlying mechanisms of microbial biogeography. Our reconstructions of microbial diversity across the natural forest ecosystems could provide a guideline for monitoring and evaluating the long-term success of the forest restoration efforts from cropland that are under way in China (50), as restoring belowground generally leads to a more successful belowground restoration (51). Meanwhile, the deviation of soil microbial communities from the corresponding natural forest state could be used to assess the extent of soil degradation. Moreover, the current microbial community states in different vegetation types could be used for predicting the long-term response of the underground ecosystem to the future changes of vegetation distribution caused by climate change. Given that the roles of microbes varied with the taxonomic group, the discrepancy of the biogeographic patterns for the three microbial taxa suggests different driving processes for various functions of the soil microbial community.

## MATERIALS AND METHODS

### Study area and sampling.

We collected three soil samples from a 100- by 100-m^2^ plot in natural, undisturbed forest at each of the 110 sites across Eastern China using a uniform sampling protocol (Fig. 1). These sites were categorized into four distinct biogeographic regions according to the classification of the vegetation regionalization map of China (http://www.nsii.org.cn/chinavegetaion) to include (i) tropical seasonal forests (TSF; 25 sites), (ii) subtropical broad-leaved evergreen forests (SBEF; 49 sites), (iii) temperate deciduous broad-leaved forests (TDBF; 17 sites), and (iv) temperate mixed coniferous-broadleaf forests (TMCF; 19 sites). Samples were collected at a depth of 0 to 15 cm after the removal of loose debris from the forest floor. Five soil cores were combined to obtain one soil sample, resulting in three analytical sample replicates per plot. All soil samples were transported to the laboratory on ice. Coarse roots and stones were removed, and a subset of the soil was air dried for analysis of edaphic properties. The methods used to obtain values for all measured edaphic variables were described in a previous study (33).

### DNA extraction, PCR, and high-throughput sequencing.

Upon the arrival of fresh soil samples at the laboratory, DNA was extracted from the soil samples using the MP FastDNA spin kit for soil (MP Biomedicals, Solon, OH) according to the manufacturer’s instructions. Equal concentrations (200 µg) of DNA extracted from the three replicates were combined to form a composite genetic pool representing total DNA for each site. DNA purity and concentration were determined using a NanoDrop spectrophotometer (NanoDrop Technologies, Inc., Wilmington, DE). Isolated total DNA was stored at −20°C for microbial diversity and sequence analyses.

We amplified a region of the 16S rRNA gene for archaea and bacteria and a region of the 18S rRNA gene for fungi using microbial tag-encoded FLX amplicon pyrosequencing (TEFAP) procedures described previously (52). The archaeal and bacterial 16S rRNA genes were amplified by primer pairs A340F90 (GYGCASCAGKCGMGAAW)/A806R96 (GGACATCVSGGGTATCTAAT) and Gray28F (GAGTTTGATCNTGGCTCAG)/Gray519R (GTNTTACNGCGGCKGCTG), respectively (33). The fungal 18S rRNA gene was amplified by primer pair funSSUF (TGGAGGGCAAGTCTGGTG)/funSSUR (TCGGCATAGTTTATGGTTAAG) (33). We used positive and negative controls throughout our experimental work. Amplified PCR products were sequenced on the 454 GS-FLX+ platform across 11 plates at the Research and Testing Laboratory (Lubbock, TX).

### Sequence analysis.

Raw sequence data were reassigned to samples in QIIME version 1.9.0 based on the barcodes and trimmed to exclude short and low-quality sequences. The following parameters were used: min_seq_length 200, max_seq_length 500, min_qual_score 25, max_homopolyer 6, truncate_ambi_bases TRUE. The remaining sequences were filtered using the denoiser_wrapper.py script with the default settings. Sequences were clustered into OTUs using the pick_open_reference_otus.py script (53) with 97% pairwise identity, using the Greengenes 16S rRNA database (release 13_8) for bacteria and archaea and the SILVA 111 database for fungi. Chimera detection was performed with the UCHIME module of USEARCH (version 8.0) (54). Putative chimeric sequences and singletons were discarded. The OTUs that were not assigned to taxa in archaea, bacteria, or fungi were removed prior to further analysis.

### Statistical analysis.

Mean annual air temperature (MAAT) and mean annual precipitation (MAP) values were obtained from the WorldClim database (http://www.worldclim.org). To reduce the bias associated with differences in library size for different samples, we normalized the microbial count data using the negative binomial model provided in the R (version 2.3.3) package *phyloseq* (55). To determine the direct and indirect effects of climatic and edaphic variables on the Shannon index, we used structural equation modeling (SEM) in the *lavaan* package (56). Model fits were explored based on root-mean-square error of approximation (RMSEA). We included potentially important variables inferred from multiple regression models and correlations to construct separate SEM models. All direct and indirect relations between exogenous and endogenous variables were tested.

Bray-Curtis similarity matrices for archaeal, bacterial, and fungal community data were calculated in *vegan*. Principal coordinate analysis was used to assess the differences in microbial communities between sites based on Bray-Curtis similarity matrices. To test for the influence of vegetation type and environmental variables on microbial communities, we used permutational multivariate analysis of variance (PERMANOVA) with Bray-Curtis matrices. Canonical correspondence analysis (CCA) was utilized to explore the relationships between bacterial and fungal communities and environmental variables, as the longest gradient lengths for preliminary detrended correspondence analysis (DCA) of the bacterial and fungal communities were all greater than three (DCA1 = 5.4 and 3.7 for bacteria and fungi, respectively). Redundancy analysis (RDA) was used to explore the relationships between the archaeal community and environmental variables, as the longest gradient length for the preliminary DCA of archaeal communities was shorter than three (DCA1 = 2.2). All nonsignificant variables were eliminated from CCA or RDA. To determine the spatial patterns of alpha- and beta-diversity, we used a kriging method to interpolate the Shannon index and the first two PCoA axes beyond sampling sites. To determine the effects of spatial distance and environmental variables on microbial communities, we carried out standard and partial Mantel tests on the Bray-Curtis distances and Euclidean distances of significant variables. All *P* values were adjusted using the Benjamini-Hochberg false discovery rate (FDR) controlling procedure (57).

To examine the influence of environmental variables on each dominant microbial OTU, a bipartite network analysis was conducted between soil properties and dominant OTUs using the maximal information coefficient in the *minerva* package in R. Dominant OTUs were identified as OTUs with relative abundances greater than 0.1%. The network was generated in the igraph package (version 1.0.1) and visualized in Gephi 0.8.2.

### Accession number(s).

Sequence data have been deposited in the public National Center for Biotechnology Information (NCBI) database under BioProject accession number PRJNA293484.
