# Supplementary material for: Distinct Biogeographic Patterns for Archaea, Bacteria, and Fungi along the Vegetation Gradient at the Continental Scale in Eastern China
Source: mSystems. 2017 Feb 7;2(1):e00174-16. doi: 10.1128/mSystems.00174-16 (PMC5296412; doi:10.1128/mSystems.00174-16)
Supplement: TABLE S1 [file sys001172083st6.docx]

| Vegetation  type | Abbreviation | Sample numbers | Latitude | Mean annual temperature (˚C) | Mean annual precipitation (mm) |
| --- | --- | --- | --- | --- | --- |
| Tropical seasonal forests | TSF | 25 | 18.8˚N~22.1˚N | 22~25 | 1000~1800 |
| Subtropical broad-leaved evergreen forests | SBEF | 49 | 22.9˚N~32.8˚N | 16~18 | 1000~2000 |
| Temperate deciduous broad-leaved forests | TDBF | 17 | 34.2˚N~42.3˚N | 8~14 | 500~1000 |
| Temperate mixed coniferous-broadleaf forests | TMCF | 19 | 42.1˚N~48.7˚N | 0~5 | 500~700 |
