# Supplementary material for: Distinct Biogeographic Patterns for Archaea, Bacteria, and Fungi along the Vegetation Gradient at the Continental Scale in Eastern China
Source: mSystems. 2017 Feb 7;2(1):e00174-16. doi: 10.1128/mSystems.00174-16 (PMC5296412; doi:10.1128/mSystems.00174-16)
Supplement: TABLE S2 [file sys001172083st7.docx]

| Variables | Axis.1 | Axis.2 | *r^2^* | *P* |
| --- | --- | --- | --- | --- |
| Mean annual air temperature (MAAT) | 0.54 | -0.84 | 0.07 | 0.02 |
| Free aluminum (Ald) | 0.73 | -0.68 | 0.05 | 0.08 |
| Mean annual precipitation (MAP) | 0.43 | -0.90 | 0.05 | 0.09 |
| Carbon:nitrogen ratio (C/N) | -0.22 | -0.98 | 0.04 | 0.15 |
| Amorphous iron:free iron ratio (Feo/Fed) | -0.76 | 0.65 | 0.04 | 0.17 |
| Humic acid (HA) | -0.31 | 0.95 | 0.03 | 0.20 |
| Total nitrogen (TN) | 0.58 | 0.81 | 0.03 | 0.23 |
| Clay proportion (Clay) | 0.36 | -0.93 | 0.03 | 0.23 |
| Soil pH (pH) | -0.12 | 0.99 | 0.03 | 0.23 |
| Humic acid:fulvic acid ratio (HA/FA) | -0.21 | 0.98 | 0.03 | 0.28 |
| Free iron (Fed) | 0.65 | -0.77 | 0.02 | 0.30 |
| Amorphous aluminum (Alo) | -0.38 | -0.93 | 0.02 | 0.39 |
| Amorphous iron (Feo) | -0.80 | -0.60 | 0.01 | 0.52 |
| Dissolved organic carbon (DOC) | 0.08 | 1.00 | 0.01 | 0.52 |
| Total dissolved nitrogen (TDN) | 0.57 | 0.82 | 0.01 | 0.54 |
| Sand proportion (Sand) | -0.01 | 1.00 | 0.01 | 0.58 |
| Available potassium (AK) | 0.97 | 0.23 | 0.01 | 0.63 |
| Silt proportion (Silt) | -0.84 | -0.54 | 0.01 | 0.74 |
| Fulvic acid (FA) | 0.16 | -0.99 | 0.01 | 0.74 |
| Organic carbon (OC) | 0.98 | 0.20 | 0.00 | 0.89 |
