# Supplementary material for: Distinct Biogeographic Patterns for Archaea, Bacteria, and Fungi along the Vegetation Gradient at the Continental Scale in Eastern China
Source: mSystems. 2017 Feb 7;2(1):e00174-16. doi: 10.1128/mSystems.00174-16 (PMC5296412; doi:10.1128/mSystems.00174-16)
Supplement: TABLE S3 [file sys001172083st8.docx]

| Variables | Axis.1 | Axis.2 | *R^2^* | *P* |
| --- | --- | --- | --- | --- |
| Amorphous iron:free iron ratio (Feo/Fed) | -1.00 | -0.05 | 0.13 | 0.00 |
| Free iron (Fed) | 0.89 | 0.46 | 0.08 | 0.01 |
| Total dissolved nitrogen (TDN) | -1.00 | -0.02 | 0.07 | 0.02 |
| Humic acid (HA) | -0.62 | -0.78 | 0.06 | 0.04 |
| Available potassium (AK) | -0.48 | 0.88 | 0.06 | 0.04 |
| Soil pH (pH) | -0.91 | 0.41 | 0.05 | 0.09 |
| Clay proportion (Clay) | 0.90 | -0.43 | 0.04 | 0.10 |
| Amorphous aluminum (Alo) | 0.49 | -0.87 | 0.04 | 0.10 |
| Free Aluminum (Ald) | 1.00 | -0.06 | 0.04 | 0.12 |
| Dissolved organic carbon (DOC) | -0.93 | 0.36 | 0.02 | 0.34 |
| Fulvic acid (FA) | 0.39 | 0.92 | 0.01 | 0.50 |
| Carbon:nitrogen ratio (C/N) | 0.09 | -1.00 | 0.01 | 0.58 |
| Amorphous iron (Feo) | -0.86 | -0.50 | 0.01 | 0.63 |
| Humic acid:fulvic acid ratio (HA/FA) | 0.06 | -1.00 | 0.01 | 0.62 |
| Total nitrogen (TN) | -0.89 | 0.46 | 0.01 | 0.71 |
| Organic carbon (OC) | -0.35 | -0.94 | 0.01 | 0.78 |
| Sand proportion (Sand) | -0.29 | 0.96 | 0.01 | 0.77 |
| Silt proportion (Silt) | -0.05 | 1.00 | 0.00 | 0.81 |
