# Supplementary material for: Distinct Biogeographic Patterns for Archaea, Bacteria, and Fungi along the Vegetation Gradient at the Continental Scale in Eastern China
Source: mSystems. 2017 Feb 7;2(1):e00174-16. doi: 10.1128/mSystems.00174-16 (PMC5296412; doi:10.1128/mSystems.00174-16)
Supplement: TABLE S4 [file sys001172083st9.docx]

| Variables | Axis.1 | Axis.2 | *r^2^* | *P* |
| --- | --- | --- | --- | --- |
| Free aluminum (Ald) | 0.81 | 0.59 | 0.16 | 0.00 |
| Mean annual precipitation (MAP) | 0.73 | 0.69 | 0.15 | 0.00 |
| Mean annual air temperature (MAAT) | 0.72 | 0.70 | 0.14 | 0.00 |
| Soil pH (pH) | -0.82 | -0.58 | 0.11 | 0.01 |
| Clay proportion (Clay) | 0.85 | 0.53 | 0.09 | 0.01 |
| Humic acid:fulvic acid ratio (HA/FA) | -0.75 | -0.66 | 0.09 | 0.01 |
| Humic acid (HA) | -0.74 | -0.68 | 0.08 | 0.01 |
| Free iron (Fed) | 0.77 | 0.64 | 0.07 | 0.02 |
| Amorphous aluminum (Alo) | 1.00 | 0.06 | 0.06 | 0.04 |
| Fulvic acid (FA) | 0.73 | 0.69 | 0.04 | 0.12 |
| Total dissolved nitrogen (TDN) | -0.83 | -0.56 | 0.04 | 0.14 |
| Dissolved organic carbon (DOC) | -0.75 | -0.66 | 0.03 | 0.18 |
| Silt proportion (Silt) | -0.46 | -0.89 | 0.02 | 0.30 |
| Total nitrogen (TN) | -0.97 | -0.23 | 0.02 | 0.31 |
| Available potassium (AK) | 0.02 | -1.00 | 0.01 | 0.59 |
| Organic carbon (OC) | -0.98 | -0.19 | 0.01 | 0.58 |
| Sand proportion (Sand) | -0.99 | 0.13 | 0.01 | 0.61 |
| Carbon:nitrogen ratio (C/N) | 0.96 | 0.27 | 0.01 | 0.72 |
| Amorphous iron (Feo) | 0.71 | 0.70 | 0.00 | 0.84 |
| Amorphous iron:free iron ratio (Feo/Fed) | -0.52 | -0.86 | 0.00 | 0.91 |
