# Supplementary figures and images for: Distinct Biogeographic Patterns for Archaea, Bacteria, and Fungi along the Vegetation Gradient at the Continental Scale in Eastern China
Source: mSystems. 2017 Feb 7;2(1):e00174-16. doi: 10.1128/mSystems.00174-16 (PMC5296412; doi:10.1128/mSystems.00174-16)

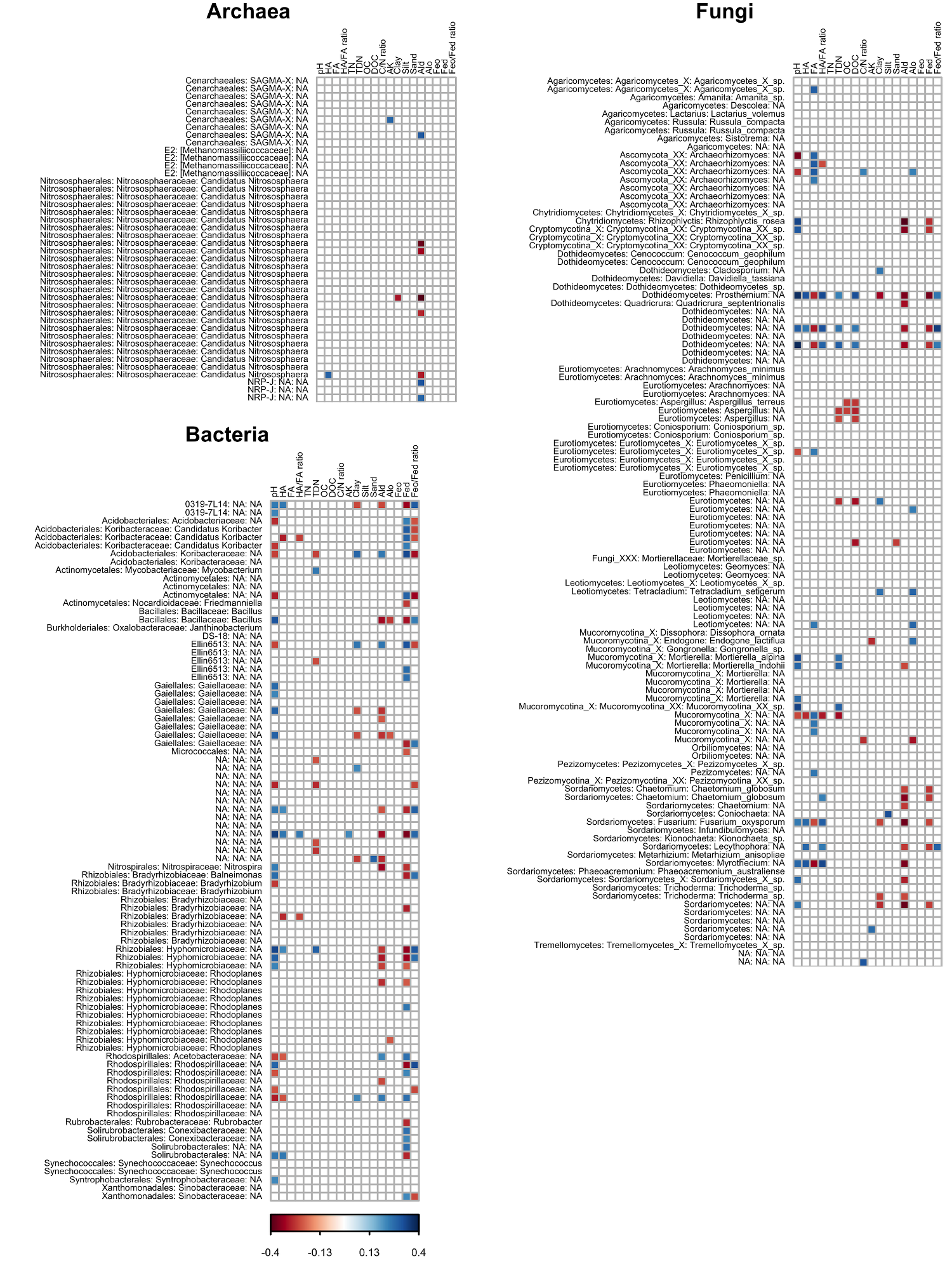

Supplement: FIG S5 [file sys001172083sf5.tif]
